# Ultrasound-Induced Modification of Durian Starch (*Durio zibethinus*) for Gel-Based Applications: Physicochemical and Thermal Properties

**DOI:** 10.3390/gels11040296

**Published:** 2025-04-16

**Authors:** Hien Vinh Nguyen, Phong Xuan Huynh, Tuyen Chan Kha

**Affiliations:** 1 Institute of Food and Biotechnology, Can Tho University, Can Tho City 94000, Vietnam; hiennv@tdmu.edu.vn (H.V.N.); hxphong@ctu.edu.vn (P.X.H.); 2 Institute of Engineering and Technology, Thu Dau Mot University, Thu Dau Mot 75100, Vietnam; 3 Faculty of Chemical Engineering and Food Technology, Nong Lam University, Ho Chi Minh City 700000, Vietnam

**Keywords:** durian starch, gelation behavior, morphological structure, physicochemical properties, rheological properties, hydrogel formation, ultrasound treatment

## Abstract

This study investigated the effects of ultrasound treatment on the physicochemical properties and thermal stability of durian starch. Durian starch samples were subjected to ultrasound at 20 kHz and 500 W for 2 min. The treatment significantly increased the starch extraction yield by 14.55% compared to untreated starch. Scanning electron microscopy analysis revealed that ultrasound treatment induced physical modifications in the starch granules, including the formation of cracks and pores, which likely contributed to the enhanced extraction efficiency and influenced the starch’s gelation behavior. Thermal analysis, including differential scanning calorimetry and thermogravimetric analysis, demonstrated that ultrasound-treated starch exhibited higher thermal stability compared to native starch. The thermogravimetric analysis results indicated a lower weight loss at high temperatures (70.39% for ultrasound-treated starch versus 79.55% for native starch at 596 °C). The heat flow during thermal decomposition was reduced in ultrasound-treated starch, suggesting that the treatment induced structural modifications that strengthened the gel matrix and improved resistance to thermal degradation. Additionally, ultrasound treatment enhanced the functional properties of durian starch, including swelling power, solubility, and water absorption capacity, which are critical for hydrogel formation and food-grade gel applications. These findings highlight the potential of ultrasound-treated durian starch for advanced applications in food hydrogels, biodegradable films, and gel-based delivery systems.

## 1. Introduction

Durian (*Durio zibethinus*) seeds are an abundant agricultural by-product, accounting for approximately 20–25% of the total fruit weight [1]. Despite being commonly discarded, they contain a high starch content (40.0–76.7%, depending on the cultivar), making them a potential raw material for starch extraction and utilization [2]. Durian seed starch has demonstrated promising applications in both food and non-food industries, including its use as a thickener, biopolymer precursor, and binder in biodegradable films. However, conventional starch extraction methods often require extensive solvent use, prolonged processing time, and result in low recovery efficiency.

Our recent report determined the optimal conditions for durian seed starch extraction: with a water-to-flour ratio of 30:1 (*w*/*w*) at pH 11, and the extraction process conducted at ambient temperature (30 °C) for 1.5 h. Ultrasonic treatment at a power of 500 W for 2 min yielded the highest starch recovery, reaching 68.13%. The results also indicated that this method achieved a higher starch yield compared to conventional extraction techniques. Additionally, durian seed starch was found to be a viable partial replacement for cassava starch in snack formulations [3].

Ultrasound-assisted extraction (UAE) has emerged as a promising green technology that enhances mass transfer, reduces processing time, and minimizes structural degradation. Previous studies on starches from various botanical sources, such as rice and maize, have demonstrated that ultrasound treatment can improve extraction yield, alter granule morphology, and modify physicochemical properties. The underlying mechanism is attributed to cavitation effects, which generate shear forces, disrupt granule structures, and facilitate starch release. Furthermore, ultrasound treatment has been reported to influence thermal transitions, lowering the gelatinization temperature, and modifying crystallinity, thereby enhancing the functional properties of starch for industrial applications.

Despite the growing interest in UAE, its impact on non-traditional starch sources, such as durian starch, remains underexplored. This study aimed to investigate the effects of ultrasound treatment on the physicochemical and thermal properties of durian starch. By evaluating parameters, including starch yield, granule morphology, crystallinity, swelling behavior, and gelatinization characteristics, this research seeks to determine the feasibility of ultrasound treatment as an efficient starch extraction method while enhancing the functional properties of durian starch. The findings could contribute to sustainable waste valorization and expand the industrial applications of durian starch.

## 2. Results and Discussion

### 2.1. The Effects of Ultrasound Power on Starch Yield

**Figure 1:**
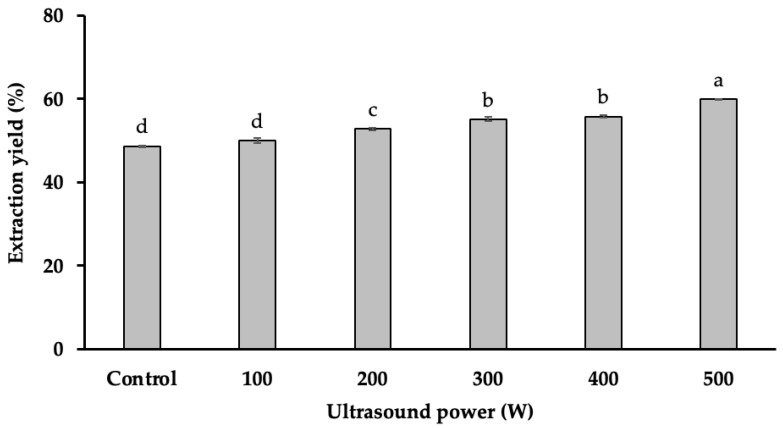
The impact of ultrasound power on starch yield. Different letters (a, b, c, d) within the same column indicate statistically significant differences (*p* < 0.05).

The results (Figure 1) indicate that starch yield was significantly influenced by ultrasound power (*p* < 0.001), showing a direct proportional relationship. The highest starch yield (59.91%) was achieved at an ultrasound power of 500 W, whereas the lowest yield (48.60%) was observed in the absence of ultrasound treatment. This represents an 11.31% increase in starch extraction efficiency when ultrasound was applied at 500 W.

The observed increase in starch yield can be attributed to the cavitation effect induced by ultrasound, which generates localized high-energy microbubbles. These bubbles collapse near the starch granules, creating intense shear forces that break down cell walls and enhance mass transfer. This disruption facilitates the release of bound starch, thereby improving extraction efficiency. Additionally, ultrasound treatment may induce microstructural modifications in starch granules, such as surface erosion and the formation of pores, which could further enhance the extraction process.

Compared to previous studies, the current findings confirm that UAE achieves higher starch extraction efficiency than conventional methods, reinforcing the advantages of this approach. For instance, a study on durian seeds found a significantly lower starch yield (26.58%) when no ultrasound treatment was applied [4], highlighting the impact of ultrasound in improving starch recovery. However, differences in starch yield can be influenced by factors such as extraction conditions, ultrasound frequency, and botanical variations. According to Baraheng and Karrila (2019), starch content varies among durian cultivars due to differences in genetic traits, growing conditions, and agronomic practices [5]. These factors, along with variations in the starch granule structure, may contribute to inconsistencies in extraction efficiency across different studies.

Despite its benefits, the application of high-intensity ultrasound requires careful optimization. Excessive ultrasound exposure or prolonged treatment duration may lead to starch degradation, reducing its functional properties. Future studies should explore the influence of different ultrasound parameters (i.e., duration) on starch quality and assess the trade-offs between yield enhancement and structural integrity. Additionally, integrating UAE with other green extraction techniques could further improve starch recovery while maintaining desirable physicochemical properties.

### 2.2. The Effects of Ultrasound Time on Starch Yield

**Figure 2:**
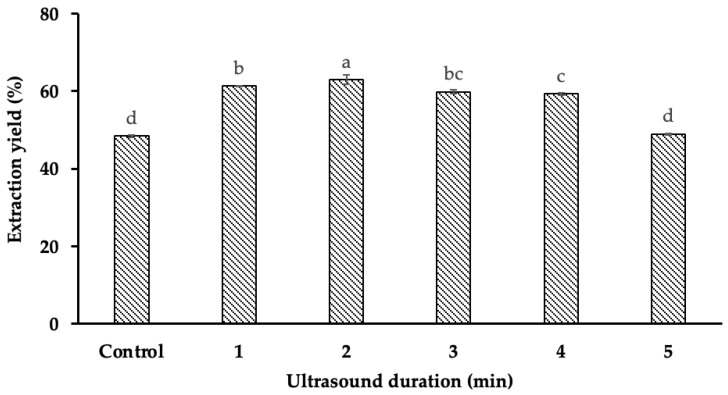
The impact of ultrasound duration on starch yield. Different letters (a, b, c, d) within the same column indicate statistically significant differences (*p* < 0.05).

The results (Figure 2) indicate that ultrasound duration significantly influenced starch yield (*p* < 0.001). Specifically, at an ultrasound duration of 2 min and a power of 500 W, the highest starch yield of 63.03% was obtained, whereas the lowest yield (48.48%) was observed in the absence of ultrasound treatment. This corresponds to a 14.55% increase in extraction efficiency when UAE was applied compared to the non-assisted method.

The enhancement in starch yield with increasing ultrasound duration can be attributed to the intensified cavitation effect. The continuous generation and collapse of microbubbles in the extraction medium create strong shear forces that disrupt the cellular structure, breaking down the mucilaginous matrix and facilitating starch release. Similar trends have been observed in previous studies. For instance, Liu et al. (2021) reported an 11% increase in corn starch yield when ultrasonic treatment (15 min, 200 W) was applied during extraction [6,7]. This suggests that ultrasound promotes starch liberation by mechanically weakening the protective polysaccharide network surrounding the granules.

Additionally, Bernardo et al. (2018) explained that durian seed starch is embedded within a dense mucilaginous matrix primarily composed of gum, which acts as a physical barrier to starch release [8]. The application of ultrasound effectively disrupts these polysaccharide structures, improving mass transfer and extraction efficiency. However, beyond an optimal duration (2 min in this study), a decline in starch yield was observed. This can be explained by the thermal effects associated with prolonged ultrasound exposure. When ultrasound time exceeded 2 min, the solvent temperature increased [9], leading to partial starch gelatinization. This gelatinization causes starch granules to swell and form a viscous mixture with the surrounding gum, thereby hindering the separation process and reducing extraction efficiency [10].

These findings highlight the importance of optimizing ultrasound parameters to maximize starch recovery without compromising its structural integrity. Future research should investigate the combined effects of ultrasound time, power, and solvent temperature to establish a balance between enhanced extraction efficiency and minimal structural degradation. Furthermore, alternative strategies, such as pulsed ultrasound application or temperature-controlled sonication, could be explored to mitigate heat-induced gelatinization while maintaining high starch yield.

### 2.3. The Impact of Ultrasound Power on the Morphology of Starch Granules

**Figure 3:**
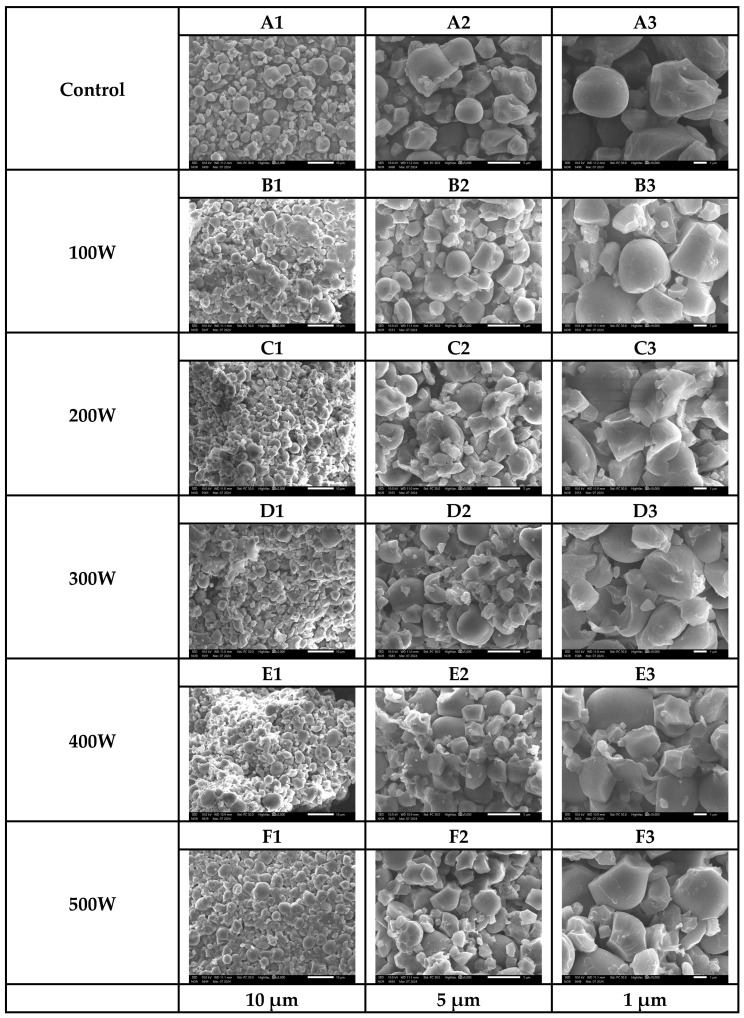
SEM images of the control (**A1**–**A3**) and ultrasound-treated samples at 100 W (**B1**–**B3**), 200 W (**C1**–**C3**), 300 W (**D1**–**D3**), 400 W (**E1**–**E3**), and 500 W (**F1**–**F3**).

The SEM images (Figure 3) reveal that native starch granules (control) exhibit diverse shapes, including spherical, cuboidal, circular, and polygonal forms, with smooth edges and intact surfaces. However, ultrasound treatment induces significant morphological changes, with the degree of structural modification directly correlated with ultrasound power. As ultrasound power increases, starch granules develop surface roughness, cracks, and, at higher power levels, substantial fragmentation. These structural alterations suggest that the mechanical forces exerted by ultrasound waves play a crucial role in modifying the granule morphology, affecting their physical integrity and functional properties.

This observation aligns with the findings from previous studies, particularly those of Jambrak et al. (2010) [9], which suggest that ultrasound-induced starch degradation primarily results from the intense shear forces exerted by cavitation. The rapid formation and collapse of microbubbles generate high-pressure gradients and violent microjets, which disrupt starch granules, creating cracks and roughened surfaces. As ultrasound power increases, these mechanical forces intensify, contributing to a reduction in the consistency coefficient, indicative of weakened structural integrity. The pronounced alterations in starch morphology and granule size observed at higher ultrasound intensities further confirm that cavitation-induced shear forces significantly influence the degradation process.

The SEM images illustrate these progressive morphological changes in starch granules subjected to different ultrasound power levels. In the control group (A1–A3), native starch granules appear intact, with smooth surfaces and well-defined shapes, indicating minimal structural disruption. At a low ultrasound power of 100 W, granules begin to exhibit slight surface roughness and small cracks (B1–B3), suggesting the initial effects of cavitation without substantial structural damage. As the ultrasound power increases to 200 W and 300 W, surface roughness becomes more pronounced, and granules show increased cracking and partial fragmentation (C1–C3, D1–D3). The mechanical forces exerted by cavitation at these power levels appear sufficient to break intermolecular interactions, particularly between starch and protein, which can alter the physicochemical properties of the granules.

At higher ultrasound powers, particularly at 400 W, deeper cracks and significant structural damage emerge (E1–E3), indicating that the cavitation effect is intensified. The collapse of microbubbles generates strong localized turbulence, accelerating granule disintegration and further compromising starch integrity. At the maximum ultrasound power of 500 W, the granules exhibit extensive rupture and collapse (F1–F3), signifying the extreme pressure gradients and microstreaming forces acting on the starch structure. At this intensity, the granule modification surpasses controlled structural changes, leading to excessive fragmentation. While ultrasound treatment is beneficial in enhancing extraction efficiency, excessive power levels may cause over-fragmentation, resulting in unintended consequences that impact starch functionality and processing behavior.

The excessive breakdown of starch granules at high ultrasound intensities may lead to increased starch loss, as the damaged granules become more soluble and dissolve into the surrounding gum matrix, complicating separation processes and ultimately reducing yield [11]. Additionally, the physical degradation of starch granules can alter their functional properties, particularly their gelatinization behavior, solubility, and retrogradation tendencies, which may affect industrial applications [12]. Over-fragmentation may also introduce challenges in downstream processing, such as filtration and drying, potentially necessitating additional processing steps to recover intact starch fractions. These factors underscore the importance of optimizing ultrasound parameters to achieve enhanced extraction efficiency while minimizing structural damage to starch granules.

Future research should explore strategies to mitigate excessive granule damage while maintaining high extraction efficiency. Pulse mode ultrasound treatments may be investigated as a means of reducing prolonged cavitation effects while still enhancing extraction. Controlled temperature sonication could help prevent excessive granule degradation and limit unintended structural alterations. Additionally, detailed physicochemical analyses are necessary to examine the effects of ultrasound power on starch functionality, including its swelling power, pasting properties, and enzymatic susceptibility. A deeper understanding of these modifications will be essential for tailoring ultrasound-assisted processing techniques to various food and industrial applications.

These findings emphasize the importance of optimizing ultrasound power to balance enhanced extraction efficiency with controlled granule modification. Future studies should explore whether pulse mode ultrasound or controlled temperature sonication could mitigate excessive granule damage while maintaining high extraction yields. Furthermore, analyzing the physicochemical changes in starch treated at different power levels can provide deeper insight into how ultrasound alters starch functionality for various applications.

### 2.4. Thermal Properties

**Figure 4:**
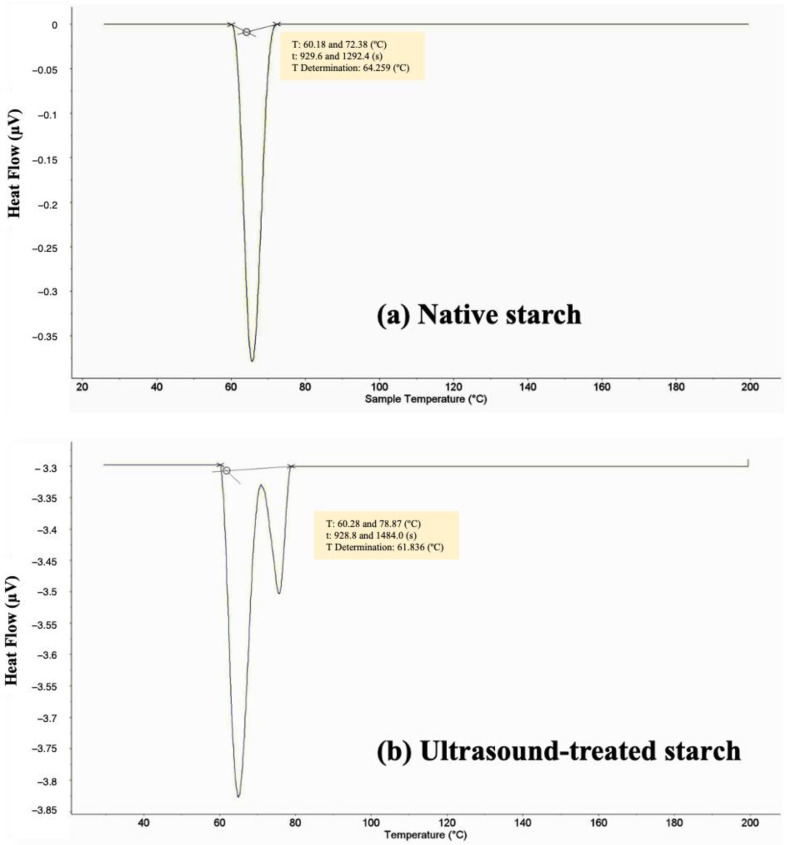
The DSC thermograms of native durian starch (**a**) and ultrasound-treated durian starch at 500 W (**b**).

The thermal properties of Kho Qua Xanh durian starch were analyzed using differential scanning calorimetry (DSC) for both native starch and starch treated with ultrasound at 500 W. The DSC thermograms are presented in Figure 4. The DSC analysis of native durian starch (Figure 4a) revealed an onset temperature (To) of 60.18 °C, a peak temperature (Tp) of 64.25 °C, an end temperature (Te) of 72.38 °C, and a gelatinization temperature range (ΔT = Te − To) of 12.2 °C. These findings align with previous studies, such as Tongdang (2008), who reported comparable values for durian seed starch, with an onset temperature of 64.43 °C, a peak temperature of 70.56 °C, and an end temperature of 75.44 °C, yielding a transition range of 11.01 °C [13].

Comparisons between durian seed starch and cassava starch indicate notable thermal differences. Cassava starch exhibits an onset temperature of 55.7 °C, a peak temperature of 65.7 °C, and an end temperature of 76.5 °C, with a broader gelatinization range of 20.8 °C. In contrast, durian seed starch demonstrates a higher onset temperature (+4.48 °C), a slightly lower peak temperature (−1.44 °C), and a lower end temperature (−4.12 °C). Furthermore, the narrower gelatinization range (ΔT = 12.2 °C) in durian starch compared to cassava starch (20.8 °C) [14] suggests a more homogenous granule population with more uniform thermal properties. This characteristic may influence the functional behavior of durian starch in food applications, particularly in processes requiring controlled gelatinization.

The DSC thermogram of ultrasound-treated starch at 500 W (Figure 4b) exhibits significant thermal modifications. The onset temperature (To) increases slightly to 60.28 °C, while the peak temperature (Tp) remains unchanged at 64.25 °C. However, the end temperature (Te) shifts markedly to 78.87 °C, extending the gelatinization range (ΔT) to 18.59 °C, which is an increase of 6.39 °C compared to the native starch. This notable shift suggests that ultrasound treatment alters the structural organization of the starch granules, potentially due to increased molecular interactions or modifications in the crystallinity of starch regions.

The increase in the gelatinization temperature range implies that ultrasound treatment enhances the thermal stability of durian starch. The cavitation and shear forces generated during sonication may disrupt the amorphous regions of the starch granules, leading to rearrangements within the crystalline domains. This structural reorganization may result in a more resistant starch network, requiring higher thermal energy to complete the gelatinization process. Furthermore, ultrasound-induced changes in granule morphology, such as increased surface roughness and fragmentation, may alter water absorption patterns, further influencing thermal properties and affecting gel formation and viscoelastic properties [11,12]. These findings highlight the potential of ultrasound treatment in tailoring starch-based hydrogels for food and biomedical applications.

These findings have critical implications for the functional application of durian starch in food processing. The observed increase in gelatinization temperature suggests that ultrasound-treated starch may exhibit enhanced thermal stability, making it suitable for high-temperature applications such as bakery products, thickening agents, and edible gels. In baked goods like snacks or pastries, thermally stable starch helps retain structural integrity, sensory quality, and nutritional value during processing at temperatures typically ranging from 150 to 250 °C. Additionally, ultrasound-induced modifications, such as granule fragmentation and increased surface roughness, may improve the viscoelastic properties and water binding capacity of starch-based systems, contributing to better texture and overall functional performance. For instance, the addition of microcrystalline cellulose to cassava starch raised the TGA peak temperature from 280 °C to 310 °C, improving its heat resistance in baking applications [15,16]. Similarly, biodegradable films composed of polylactic acid and nano-chitosan demonstrated an increase in decomposition temperature from 305 °C to 330 °C, indicating enhanced thermal resilience suitable for hot-extrusion processing [17,18].

However, the broader gelatinization transition range observed in ultrasound-treated starch may be reflected in the increased molecular heterogeneity due to partial disruption of crystalline regions and rearrangement within the amorphous matrix. Ultrasound treatment generates localized shear forces and cavitation, which can disturb the internal structure of starch granules, resulting in a less uniform crystalline architecture and a higher proportion of amorphous regions. This structural heterogeneity causes granules to exhibit varying thermal stabilities, leading to a wider range of gelatinization temperatures. Changes in the amorphous-to-crystalline ratio have been widely associated with such thermal behavior, as previously reported in modified starch systems. Therefore, further research is warranted to explore whether the use of controlled ultrasound parameters, such as pulsed ultrasound or moderate energy input, can minimize granule heterogeneity and optimize both the thermal and functional properties of starch for industrial applications.

### 2.5. Thermogravimetric Analysis

**Figure 5:**
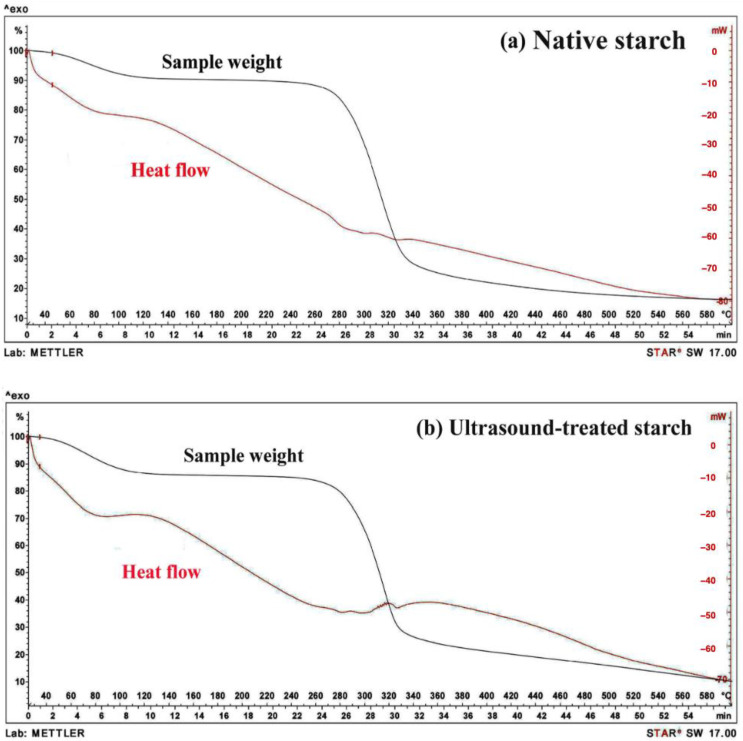
TGA thermograms of native durian starch (**a**) and ultrasound-treated durian starch (500 W) (**b**) as a function of temperature and time.

Thermogravimetric analysis (TGA) was conducted to evaluate the thermal stability and weight loss of durian starch samples as the temperature increased. The TGA results for native durian starch are presented in Figure 5a, while the analysis of ultrasound-treated starch (500 W for 2 min) is shown in Figure 5b.

#### 2.5.1. Thermal Decomposition of Native and Ultrasound-Treated Starch

The TGA of native durian starch revealed three distinct phases of mass reduction, each corresponding to different stages of thermal decomposition. In the first phase (25 °C to 260 °C), the starch exhibits a gradual weight loss, primarily attributed to the evaporation of moisture, and the volatilization of light organic compounds. The second phase (260 °C to 340 °C) is characterized by a more substantial weight reduction, which is linked to the thermal degradation of hemicellulose and cellulose. The third phase (340 °C to 600 °C) corresponds to the decomposition of lignin. These findings are consistent with the general thermal degradation patterns observed in plant-based starches, wherein hemicellulose decomposes first, followed by cellulose, and finally, lignin, which requires higher temperatures for degradation [19]. Similar trends have been reported in previous studies by Grønli et al. (2002) and Demirbas (2004), who highlighted the stepwise decomposition behavior of biomass-derived materials [20,21].

A critical comparison of the TGA curves for native and ultrasound-treated starch (Figure 5) revealed notable differences in thermal stability, which have significant implications for starch-based gel applications. The native starch exhibits greater weight loss than the ultrasound-treated starch, indicating lower thermal resistance. Specifically, at 596 °C, the native starch undergoes a weight reduction of 79.55%, whereas the ultrasound-treated starch loses only 70.39%. The lower weight loss in ultrasound-treated starch suggests enhanced stability, likely due to structural modifications induced by ultrasonic cavitation. These changes may influence the gelation behavior of starch, improving its suitability for thermally stable hydrogel formulations and functional applications requiring enhanced resistance to high-temperature processing.

#### 2.5.2. Heat Flow Analysis and Implications

The DSC curves (shown in red in Figure 5) provide additional insight into the thermal behavior of the starch samples. At 596 °C, the native starch generates a heat flow of 16.1899 mV, which is substantially higher than that of the ultrasound-treated starch (10.2330 mV). The increased heat flow in native starch suggests that its thermal degradation is more exothermic, possibly due to a greater release of volatile compounds during decomposition. Interestingly, in the temperature range of 300 °C to 500 °C, the ultrasound-treated starch exhibits a higher heat flow than the native starch, indicating potential alterations in its thermal transition mechanisms.

The findings suggest that ultrasound treatment enhances the thermal resistance of starch by modifying its structural organization. These modifications may involve the rearrangement of starch granules, increased cross-linking, or alterations in crystallinity, leading to a more thermally stable matrix. Furthermore, the reduction in heat flow indicates that ultrasound treatment may limit the release of energy-intensive volatile compounds, potentially influencing the gelatinization, retrogradation, and viscoelastic behavior of starch in hydrogel or composite gel applications [11,22]. These insights provide a foundation for developing ultrasound-modified starch as a functional component in thermally stable gels for industrial applications.

#### 2.5.3. Quantitative Analysis of Thermal Stability

The thermal stability characteristics of both starch types are summarized in Table 1, highlighting key parameters such as sample weight, heat flow, and percentage weight loss at 596 °C. The data further reinforce the claim that ultrasound-treated starch exhibits superior thermal stability, as evidenced by its lower weight loss and reduced heat flow at elevated temperatures. The structural modifications induced by ultrasound may contribute to stronger molecular interactions, making the starch more resistant to thermal degradation.

#### 2.5.4. Implications for Food Processing and Industrial Applications

The observed improvement in thermal stability of ultrasound-treated starch has significant implications for food processing and industrial applications that require starch with enhanced heat resistance. In food technology, starches with greater thermal stability are highly desirable for applications involving high-temperature processing, such as extrusion, baking, and frying. The ability of ultrasound treatment to alter the thermal degradation pathway of starch could provide new opportunities for tailoring starch functionality to specific food formulations.

From a physicochemical perspective, ultrasound treatment may induce structural modifications such as increased molecular ordering, enhanced cross-linking, or changes in granule morphology, all of which could contribute to improved thermal properties [11,12]. However, further investigations are necessary to elucidate the exact mechanisms underlying these changes. Future studies should focus on analyzing crystallinity, molecular weight distribution, and gelatinization behavior using techniques such as X-ray diffraction (XRD), Fourier transform infrared spectroscopy (FTIR), and rheological analysis.

Moreover, the application of ultrasound technology to modify starch properties could extend beyond food science into biodegradable packaging, pharmaceuticals, and biomedical applications, where thermal stability plays a crucial role in material performance. The ability to fine-tune starch properties using non-chemical methods, such as ultrasound, offers a sustainable and environmentally friendly alternative to traditional starch modification techniques that rely on chemical additives.

In conclusion, the thermogravimetric analysis demonstrates that ultrasound treatment significantly enhances the thermal stability of durian starch, as reflected in the lower weight loss and reduced heat flow at higher temperatures compared to native starch. The structural modifications induced by ultrasound may contribute to enhanced heat resistance, making the starch more suitable for high-temperature applications in the food and non-food industries. Future research should aim to elucidate the molecular-level changes responsible for these improvements and explore the broader implications of ultrasound treatment in gel-based materials, biodegradable films, and functional food development.

### 2.6. The Effect of Ultrasound on Viscosity

Viscosity is a fundamental rheological property of starch that significantly influences the texture, stability, and functionality of starch-based products. The viscosity of starch dispersions is primarily governed by the interaction between starch molecules and water, wherein the hydroxyl groups in starch form intermolecular hydrogen bonds, promoting molecular aggregation and water retention [23]. The degree of these interactions directly affects the swelling behavior, gelatinization process, and overall viscosity of the starch suspension [24].

**Figure 6:**
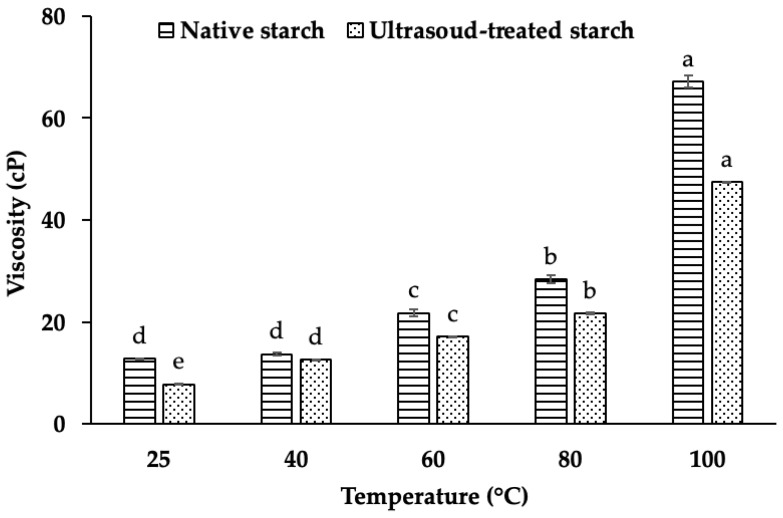
Viscosity of native starch and ultrasound-treated starch at different temperatures. Different letters (a, b, c, d, e) within the same column indicate statistically significant differences (*p* < 0.05).

A comparison of native and ultrasound-treated starch demonstrates a statistically significant reduction in viscosity following ultrasound treatment (*p* < 0.001). Notably, the viscosity of native starch is 24.21% higher than that of ultrasound-treated starch at the same temperature (Figure 6). This substantial decrease suggests that ultrasound-induced modifications alter the structural integrity of starch granules, likely affecting their hydration capacity and gel-forming ability.

The viscosity of starch suspensions increases significantly within the temperature range of 60–100 °C, consistent with the well-documented gelatinization process. This phenomenon is attributed to the swelling of starch granules and the subsequent leaching of amylose into the surrounding medium, which collectively enhance the viscosity of the paste [25]. At temperatures below the gelatinization threshold, starch granules remain largely intact, and their hydration capacity is limited, resulting in lower viscosity. However, as the temperature rises, the starch granules absorb water, swell, and undergo molecular rearrangement, thereby increasing viscosity [24].

Thiré et al. (2003) reported that starch granules generally remain undamaged at temperatures below the gelatinization temperature, which contributes to the viscosity increase in native starch suspensions [26]. However, when starch is subjected to ultrasound treatment, the disruption of hydrogen bonding weakens granule integrity, leading to a reduction in swelling capacity and viscosity. This suggests that ultrasound-induced structural modifications interfere with the ability of starch molecules to retain water, thereby altering its rheological behavior.

The reduction in viscosity following ultrasound treatment is primarily due to hydrogen bond disruption, granule fragmentation, molecular depolymerization, and decreased water absorption capacity. Ultrasound generates cavitation forces, which weaken intermolecular hydrogen bonding in starch molecules, reducing molecular entanglement and limiting viscosity. Additionally, the mechanical shear stress induced by ultrasound causes granule fragmentation and partial depolymerization of amylopectin, lowering molecular weight and reducing the starch’s ability to retain water. This structural modification weakens the swelling capacity of starch granules, further limiting viscosity increases at higher temperatures [11,12,22].

Moreover, ultrasound-treated starch exhibits enhanced shear-thinning behavior, making dispersions more fluid and less resistant to deformation. This characteristic is beneficial for applications requiring improved flow properties, such as the use of low-viscosity starches in beverages, gels, emulsions, and 3D-printed food products. Overall, ultrasound treatment alters starch’s molecular structure, enhancing its processability in both food and industrial applications. Future research should further explore these structural changes using techniques such as XRD, FTIR, and NMR spectroscopy to elucidate the underlying physicochemical transformations involved in gelation behavior and network formation.

### 2.7. Physicochemical Properties of Native and Ultrasound-Treated Starches

Ultrasound treatment significantly enhanced water absorption capacity (WAC), swelling power, and ash content, while water content decreased, and color remained unchanged (Table 2). The increase in WAC and swelling power suggests structural modifications within the starch granules, likely due to the disruption of the crystalline and amorphous regions caused by ultrasound-induced cavitation. According to Rahaman et al. (2021), ultrasonic treatment can disrupt the structure of starch granules and other biopolymers, leading to amorphization, the transformation from an ordered to a disordered structure [27]. This structural disruption increases void volume and porosity, facilitating greater water penetration. Similarly, Jambrak et al. (2010) reported that cavitation induced by ultrasound leads to the collapse of microbubbles, generating localized high temperatures and pressures [9]. These conditions break hydrogen bonds and disrupt crystalline regions, converting them into amorphous forms. Amorphous regions are highly hydrophilic and swell readily in aqueous environments, thereby enhancing the water permeability of starch-based films or gels [28].

According to Jambrak et al. (2010), ultrasound disrupts starch granules by weakening molecular interactions, allowing for greater water penetration and retention, which is crucial for hydrogel formation and gel-based applications [9]. In addition, ultrasound can induce the release of amylose from starch granules due to the breakdown of the granule structure and the disruption of bonds that confine amylose. The released amylose may contribute to increased viscosity and gelling capacity in aqueous systems through the formation of molecular networks, which also influence water permeability properties [29]. The higher ash content in ultrasound-treated starch may result from enhanced solubility and dispersion of minerals during processing, potentially influencing the ionic interactions within gel networks. Furthermore, the reduction in water content indicates improved dehydration efficiency, which can be advantageous in industrial applications requiring lower moisture levels, such as dried gels and gel-based stabilizers. These findings suggest that ultrasound treatment enhances starch functionality, making it more suitable for applications where increased hydration, swelling capacity, and controlled gelation properties are beneficial. Further structural analysis could provide deeper insights into the extent of granule disruption and crystallinity changes, shedding light on its role in supramolecular gel systems.

## 3. Conclusions

This study demonstrates that ultrasound treatment at 500 W for 2 min significantly enhances the physicochemical, gelation, and thermal properties of durian starch. Structural modifications, including the formation of cracks and pores, contributed to increased swelling power, solubility, water absorption capacity, and improved gel-forming ability. Thermal analysis further revealed enhanced stability, with ultrasound-treated starch exhibiting lower weight loss (70.39%) and reduced heat flow at 596 °C compared to untreated starch (79.55%). These findings suggest that ultrasound treatment improves starch’s resistance to thermal degradation and modulates its gelation behavior, making it suitable for applications in hydrogels, edible films, and functional food formulations. Future research should explore its potential in structuring gels for biomedical and industrial applications, as well as the economic feasibility of applying ultrasound-treated durian starch.

## 4. Materials and Methods

### 4.1. Durian Seed

Kho Qua Xanh durian seeds used in this study were obtained from processing facilities in Cai Lay, Tien Giang, Vietnam [30,31]. The seeds were peeled, thinly sliced, and dried using hot air at 50 °C for 48 h until reaching a moisture content of approximately 6%. The dried seeds were then ground using a milling machine, sieved through a 0.3 mm mesh, and packaged in 0.5 kg plastic bags. The samples were stored at 4–6 °C until further use.

### 4.2. Starch Extraction

Durian seed flour (5 g) was mixed with distilled water containing 1% NaCl at a flour-to-water ratio of 1:30 (*w*/*w*) [3,32,33] and subjected to ultrasound treatment using a VCX 500 ultrasonic processor (Sonics & Materials, Inc., Newtown, CT, USA) equipped with a standard probe (139 mm in length) and a replaceable tip (13 mm in diameter). The treatment was performed at a constant frequency of 20 kHz, with varying powers and durations. During ultrasound treatment, the sample temperature was maintained at 30 °C using a water bath. Following sonication, the mixture was incubated at 30 °C for 90 min, and subsequently centrifuged at 4000 rpm for 10 min. The resulting starch precipitate was washed with a 0.05% Na_2_S_2_O_5_ solution, dried at 40 °C for 24 h, ground into a fine powder, and weighed to determine the physicochemical and thermal properties.

### 4.3. Analyses

#### 4.3.1. Moisture Content

The moisture content of the samples was determined by drying in a laboratory air oven at 105 °C until a constant weight was achieved (925.09, AOAC 1990).

#### 4.3.2. Ash Content

The ash content was measured following the standard AOAC method (923.03, AOAC 1990).

#### 4.3.3. Scanning Electron Microscopy Analysis

The morphology, surface structure, and granule size of durian seed starch were analyzed using a scanning electron microscope (JSM IT 200, JEOL, Akishima, Japan) with an acceleration voltage of 10–20 kV. SEM images were captured at magnifications ranging from 1000× to 10,000×.

#### 4.3.4. Gelatinization Temperature

Starch gelatinization was analyzed using a differential scanning calorimeter (DSC) (Thermo Analyzer Labsys Evo S60, Setaram, Villebon-sur-Yvette, France). Starch–water mixtures were prepared at a 1:4 (*w*/*w*) ratio, sealed in DSC pans, and equilibrated for 12 h. The samples were then heated from 25 °C to 200 °C at a rate of 2 °C/min. The onset temperature (T_onset_), peak temperature (T_peak_), and end temperature (T_end_) were recorded, and the gelatinization range (ΔT = T_end_ − T_onset_) was calculated [14].

#### 4.3.5. Thermogravimetric Analysis (TGA)

Thermal stability and mass loss were analyzed using a thermogravimetric analyzer (TGA/DSC 3+, Mettler Toledo, Columbus, OH, USA). The sample was dried and equilibrated prior to the TGA. The sample weight was recorded while heating from 25 °C to 600 °C over a period of 60 min in a nitrogen environment at a heating rate of 10 °C/min.

#### 4.3.6. Water Absorption Capacity

Water absorption capacity (WAC) was measured by mixing durian seed starch with distilled water at a ratio of 1:15 (*w*/*w*), vortexing for 2 min, and centrifuging at 1250× *g* for 20 min. WAC was calculated as the grams of water absorbed per gram of dried starch, following the removal of the supernatant and residual droplets [34].

#### 4.3.7. Swelling Power and Solubility

Swelling power and solubility were determined using the method of Kittipongpatana et al. (2011) [35].

Swelling Power: A 2 g sample was placed into a pre-weighed centrifuge tube containing 200 mL of water (1% *w*/*v*), vortexed for 1 min, and left to stand for 10 min. The mixture was centrifuged at 3000 rpm for 15 min. The precipitate was weighed after centrifugation.

$$Swelling\;power\;(\%)=\frac{m_{1}}{m_{2}}\times 100$$

where m_1_ is the mass of sedimented starch (g) and m_2_ is the mass of dried starch (g).

Solubility: The supernatant was transferred to a pre-weighed crucible and dried at 120 °C to a constant weight to determine solubility.

$$Solubility\;(\%)=\frac{m_{3}}{m}\times 100$$

where m_3_ is the mass of dissolved substance in the centrifuged solution (g) and m is the initial mass of the sample (g).

#### 4.3.8. Viscosity

Viscosity was measured using the method of Spychaj et al. (2013) [36]. A 10 g sample of dried and equilibrated starch was dissolved in distilled water to obtain a total weight of 500 g. The starch suspension was stabilized in a water bath for 30 min under continuous stirring. Viscosity measurements were performed using a Brookfield DV2T viscometer (Brookfield Engineering, Middleboro, MA, USA) at 200 rpm with spindle No. 2.

### 4.4. Statistical Analyses

All experiments and subsequent analyses were performed in triplicate, and the results were expressed as mean ± standard deviation. Statistical analyses were conducted using one-way analysis of variance (ANOVA) in Minitab 16 software (Minitab, LLC., State College, PA, USA) Differences between treatments, i.e., ultrasound powers and durations, were evaluated using the least significant difference (LSD) test at a 5% significance level (*p* < 0.05).

## Figures and Tables

**Table 1:** Thermal stability of native and ultrasound-treated durian starch.

Starch Type	Sample Weight (mg)	E Indium (1/mW)	Heat Flow (mV/°C)	Sample Weight (%/°C)
Native starch	11.4350	171.5119	16.1899 ^a^/596.667	−79.5530/596.667
Ultrasound-treated starch	12.4750	171.5119	10.2330 ^b^/596.667	−70.3929/596.667

Different letters (a, b) within the same row indicate statistically significant differences (*p* < 0.05).

**Table 2:** Physicochemical properties of native and ultrasound-treated starches at 500 W.

Parameters	Native Starch	Ultrasound-Treated Starch
Ash content (%)	1.93 ± 0.07 ^a^	2.14 ± 0.02 ^b^
Water content (%)	6.83 ± 0.04 ^a^	5.27 ± 0.07 ^b^
Water absorption capacity (%)	12.64 ± 0.05 ^a^	24.21 ± 0.08 ^b^
Swelling power	18.30 ± 0.02 ^a^	21.60 ± 0.04 ^b^
Color	Brownish-white	Brownish-white

Different letters (a, b) within the same row indicate statistically significant differences (*p* < 0.05).

## Data Availability

The original contributions presented in the study are included in the article, further inquiries can be directed to the corresponding author.
